# The crucial role of single-stranded DNA binding in enhancing sensitivity to DNA-damaging agents for Schlafen 11 and Schlafen 13

**DOI:** 10.1016/j.isci.2023.108529

**Published:** 2023-11-23

**Authors:** Kohei Fujiwara, Masashi Maekawa, Yuki Iimori, Akane Ogawa, Takeshi Urano, Nobuaki Kono, Hiroyuki Takeda, Shigeki Higashiyama, Makoto Arita, Junko Murai

**Affiliations:** 1 Division of Physiological Chemistry and Metabolism, Graduate School of Pharmaceutical Sciences, Keio University, Minato-Ku, Tokyo 105-8512, Japan; 2 Institute for Advanced Biosciences, Keio University, Tsuruoka, Yamagata 997-0052, Japan; 3 Laboratory for Metabolomics, RIKEN Center for Integrative Medical Sciences, Yokohama, Kanagawa 230-0045, Japan; 4 Department of Biochemistry, Faculty of Medicine, Shimane University, Izumo, Shimane 693-8501, Japan; 5 Center for Vaccines and Therapeutic Antibodies for Emerging Infectious Diseases, Shimane University, Izumo, Shimane 693-8501, Japan; 6 Systems Biology Program, Graduate School of Media and Governance, Keio University, Fujisawa, Kanagawa 252-0882, Japan; 7 Division of Proteo-Drug-Discovery, Proteo-Science Center, Ehime University, Matsuyama, Ehime 790-8577, Japan; 8 Division of Cell Growth and Tumor Regulation, Proteo-Science Center, Ehime University, Toon, Ehime 791-0295, Japan; 9 Department of Biochemistry and Molecular Genetics, Ehime University Graduate School of Medicine, Toon, Ehime 791-0295, Japan; 10 Department of Oncogenesis and Tumor Regulation, Osaka International Cancer Institute, Chuo-Ku, Osaka 541-8567, Japan; 11 Human Biology-Microbiome-Quantum Research Center (WPI-Bio2Q), Keio University, Tokyo, Japan; 12 Cellular and Molecular Epigenetics Laboratory, Graduate School of Medical Life Science, Yokohama City University, Yokohama, Kanagawa 230-0045, Japan

**Keywords:** Drugs, Biochemistry, Molecular biology

## Abstract

Schlafen (SLFN) 11 enhances cellular sensitivity to various DNA-damaging anticancer agents. Among the human SLFNs (SLFN5/11/12/13/14), SLFN11 is unique in its drug sensitivity and ability to block replication under DNA damage. In biochemical analysis, SLFN11 binds single-stranded DNA (ssDNA), and this binding is enhanced by the dephosphorylation of SLFN11. In this study, human cell-based assays demonstrated that a point mutation at the ssDNA-binding site of SLFN11 or a constitutive phosphorylation mutant abolished SLFN11-dependent drug sensitivity. Additionally, we discovered that nuclear SLFN13 with a point mutation mimicking the DNA-binding site of SLFN11 was recruited to chromatin, blocked replication, and enhanced drug sensitivity. Through generating multiple mutants and structure analyses of SLFN11 and SLFN13, we identified protein phosphatase 2A as a binding partner of SLFN11 and the putative binding motif in SLFN11. These findings provide crucial insights into the unique characteristics of SLFN11, contributing to a better understanding of its mechanisms.

## Highlights

• Single-stranded DNA binding site of SLFN11 (K652) is essential for chromatin loading
• SLFN13 with a DNA binding site (E652K) can functionally mimic SLFN11
• PP2A binding motif in SLFN11 contributes to SLFN11-dependent drug sensitivity
• SLFN11-dependent drug sensitivity is well consistent with replication block by SLFN11

## Graphical abstract

**Figure:**
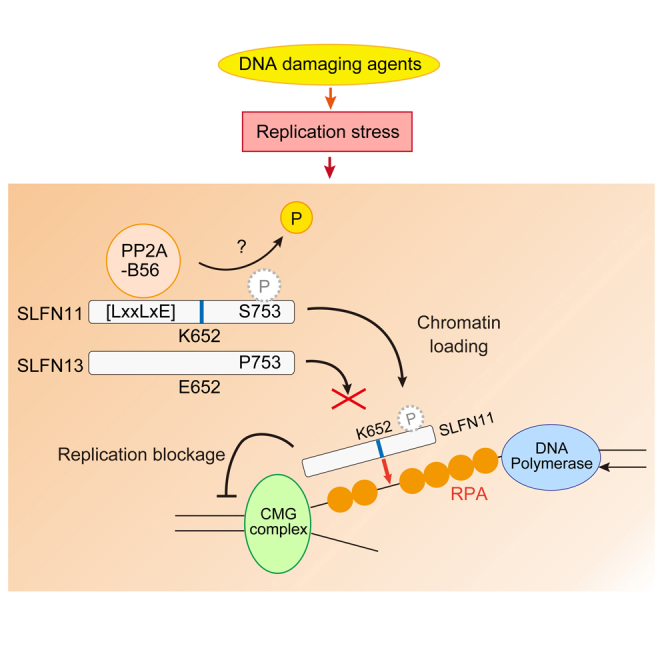

## Introduction

DNA-damaging anticancer agents (DDAs), such as platinum derivatives and topoisomerase I and II inhibitors, have been applied in the clinical setting for decades. However, the clinical application of predictive biomarkers for responses to DDAs has not yet been established. Schlafen 11 (SLFN11) has attracted attention as a candidate because its mRNA expression level is highly correlated with sensitivity to various DDAs in multiple cancer cell line databases.^1^^,^^2^ The causative relationship between elevated SLFN11 expression and high sensitivity to DDAs was subsequently validated through genetic approaches with several cell systems.^3^^,^^4^^,^^5^^,^^6^^,^^7^ Moreover, the clinical implications of SLFN11 have been validated in multiple cancers, including breast,^8^ ovary,^9^^,^^10^^,^^11^ stomach,^12^ bladder,^13^ lung,^14^^,^^15^ esophagus,^16^ medulloblastoma,^17^ head and neck,^18^ and prostate.^19^

*Slfn* genes (*Slfn1–14*) evolved rapidly after the branching of boreoeutheria.^20^ Among the 5 human SLFNs (SLFN5, 11, 12, 13, 14), SLFN5, 11, 13, and 14 commonly harbor a nuclease domain at the N-terminus, an SWAVDL motif in the middle region, and a putative helicase/ATPase domain with Walker A/B motifs at the C-terminus^21^ (Table S1). Despite the strong conservation of sequences and structures among SLFNs, SLFN11 is unique due to its highly significant correlation with sensitivity to DDAs.^22^ Although the mechanisms of SLFN11-mediated cell killing are not fully understood, two functions of SLFN11 are most likely. One is the endonuclease activity for type II tRNA that attenuates the translation of the ATR and ATM kinases, master regulators of the DNA damage response.^23^^,^^24^^,^^25^ Dephosphorylation at the N-domain (S219 and T230) and the C-domain (S753) of SLFN11 is important for SLFN11-dependent tRNA cleavage.^23^ Notably, the N-terminal nuclease active site residues E209, E214, and K216 in SLFN11 are well conserved in human SLFN11, -12, −13, and -14.^26^^,^^27^^,^^28^^,^^29^ Conversely, human SLFN5 lacks the K216 residue and has no endonuclease activity.^26^

The other is the unique function of SLFN11 that blocks replication independently of the ataxia telangiectasia and Rad3-related (ATR) activation (i.e., S-phase checkpoint) following replication stress.^7^ SLFN11 has replication protein A1 (RPA1) binding domain in the C-terminus^3^ and is recruited to replication forks via RPA1/2/3 complex (RPA)-coated ssDNA under replication stress conditions.^7^ Stressed replication forks harbor RPA-coated ssDNA gaps to which SLFN11 binds, inducing permanent replication blockage (i.e., until the cell dies). SLFN11 with a Walker B motif mutation (E669Q, a putative ATPase-dead) can still be recruited to stressed replication forks, but lacks the replication-blocking and drug-sensitizing capabilities. Recent cryo-electron microscopy and biochemical analyses have revealed that SLFN11 preferentially binds ssDNA rather than double-stranded DNA (dsDNA), in a dimeric state.^30^ The results showed that SLFN11 (K652) is the ssDNA binding site, and dephosphorylation at S753 is critical for ssDNA binding. However, the relationship between ssDNA binding and drug sensitivity remains unclear. Furthermore, the reasons why SLFN11 is unique compared with other SLFNs have not been clarified.

In this study, we searched for the domains, amino acid residues, and interactors that determine the uniqueness of SLFN11. Using human cell-based analyses, we found that the ssDNA binding capability of SLFN11 is crucial for its functions (chromatin binding, replication block, drug sensitivity). Then, we explored the possibility of converting SLFN13 into a protein resembling SLFN11 with minimal modifications. We identified protein phosphatase 2A (PP2A) as a binding partner of SLFN11 and the binding domain-associated SLFN11-dependent drug sensitivity.

## Results

### The single-stranded DNA binding site (K652) and the dephosphorylation of S753 are critical for Schlafen 11-dependent drug sensitivity

**Figure 1:**
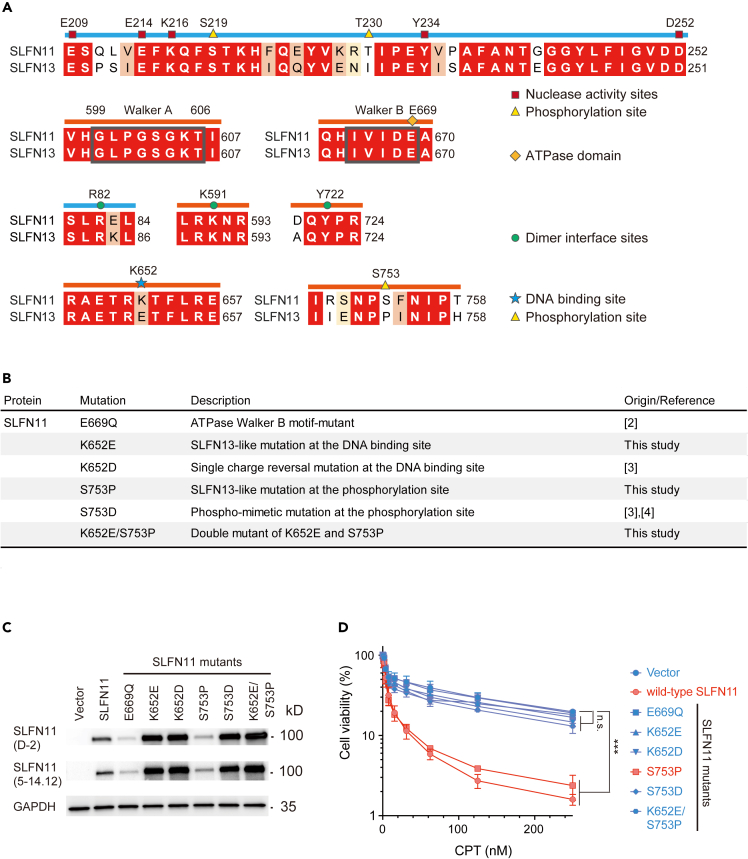
The ssDNA binding site (K652) and the phosphorylation site (S753) regulate SLFN11-dependent drug sensitivity (A) Pairwise amino acid sequence alignment. Residues are colored according to the percentage of identity (red = match, orange = more conserved, yellow = less conserved, white = mismatch). (B) List of SLFN11 mutants used in this study. (C) Representative immunoblots prepared from K562 cells expressing WT and mutant SLFN11 proteins. The indicated antibodies were used. (D) Viability curves of K562 cell lines treated with various concentrations of CPT for 72 h. Error bars represent means ± standard deviations (n = 3). ∗∗∗p < 0.0001 (Dunnett test); n.s., not significant. See also Figures S1–S3.

SLFN13 shares the highest amino acid conservation with SLFN11 (78% identity and 83% similarity; Table S2). However, in contrast to SLFN11, SLFN13 expression is not correlated with drug sensitivity, although the expression levels of both proteins vary extensively (Figure S1). Among the functional domains and sites of SLFN11, the dimer surface sites (R82, K591, Y722), nuclease activity sites (E209, E214, K216, Y234, D252), and SWAVDL, Walker A, and Walker B motifs are perfectly conserved between SLFN11 and SLFN13 (Figures 1A, S2A, and S2B). However, one ssDNA binding site (K652) and two phosphorylation sites (T230 and S753) of SLFN11 are not conserved in SLFN13 (Figure 1A). Given that S753 dephosphorylation is important for activating the ssDNA binding ability of SLFN11, we speculated that K652 and S753 in SLFN11 may serve as determinants for its unique functions. Hence, we designed various constructs expressing SLFN11 with or without mutations at K652, S753, or both (Figure 1B).

The K652E and K652D mutations eliminate the positive charge of K652. The S753P mutation mimics the constitutively dephosphorylated state, whereas S753D mimics the constitutively phosphorylated state. We generated stable cells expressing (over 80%) wild-type or mutant SLFN11 in K562 human leukemia cells, which normally have undetectable levels of endogenous SLFN11 (Figures 1C and S3A). The S753P SLFN11 construct was expressed at lower levels compared to the other SLFN11 mutants (K652E, K652D, S753D, and K652E/S753P). A cell viability assay confirmed that the S753P SLFN11 mutant conferred sensitivity to camptothecin (CPT), similar to the wild-type SLFN11, whereas the other SLFN11 mutants (K652E, K652D, S753D, and K652E/S753P) did not (Figure 1D). The differences in cell death were morphologically validated by microscopy (Figure S3B) and the detection of cleaved poly(ADP-ribose) polymerase 1 (PARP1) and cleaved caspase 3 (Figure S3C), which are both produced upon apoptosis. These results suggested that the ssDNA binding site (K652) and the dephosphorylation of S753 in SLFN11 are critical for SLFN11-dependent drug sensitivity.

### Chromatin binding is required for the functions of Schlafen 11

**Figure 2:**
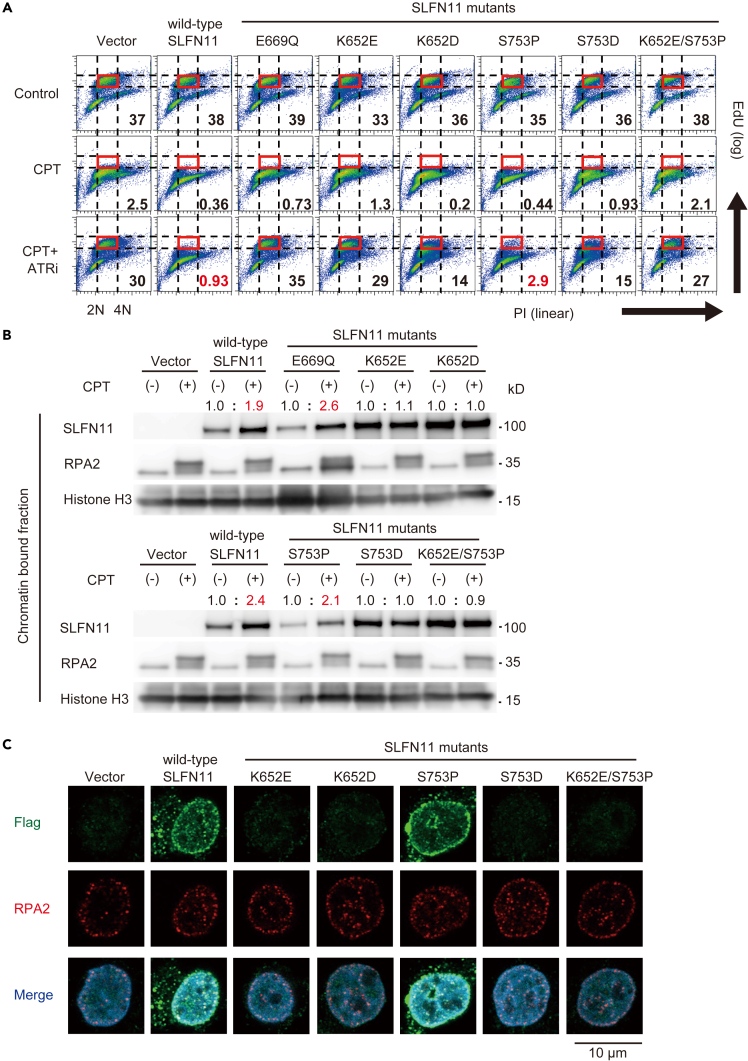
The ssDNA binding site (K652) and dephosphorylated S753 are critical for the chromatin recruitment of SLFN11 and the replication block (A) Cell cycle analysis by flow cytometry. K562 cells were treated with the indicated drugs (CPT 100 nM and ATRi 1 μM) for 4 h. The cells were labeled with EdU for 1 h before harvest. Each number represents the percentage of highly replicating cells in the red box. Data are representative of two independent experiments. ATRi: AZD6738, EdU: 5-ethynyl-2′-deoxyuridine. (B) Representative immunoblots of the chromatin-bound fractions from K562 cells expressing the indicated genes. Cells were treated for 4 h with 250 nM CPT. RPA2 was used as a positive control because CPT increases its binding to chromatin. Numbers represent signal intensity ratios of SLFN11 expression normalized to histone H3 intensity between the control and CPT-treated groups. Red values indicate increased signal intensities. (C) Representative immunofluorescence confocal microscopy images of chromatin-bound Flag-tagged SLFN11 (green), RPA2 (red), and DAPI (blue) in K562 cells expressing the indicated genes. Cells were treated with 100 nM CPT for 4 h. See also Figure S4. See also Figure S4.

The SLFN11-dependent replication block is a hallmark of its functionality.^7^ To examine the effects of the SLFN11 mutants, we performed an EdU-labeled cell cycle analysis. As expected, CPT suppressed replication regardless of SLFN11 because it activates ATR (S-phase checkpoint) (Figure 2A, compare Control vs. CPT). The addition of an ATR inhibitor restored the replication in SLFN11-negative cells (Figure 2A, Vector), but exhibited little efficacy in wild-type SLFN11-expressing cells (Figure 2A, wild-type SLFN11). The S753P SLFN11 mutant maintained the replication block, whereas the other SLFN11 mutants (E669Q, K652E, K652D, S753D, and K652E/S753P) restored replication under the CPT+ATR inhibitor treatment conditions (Figure 2A).

Next, to examine the chromatin binding capabilities of these SLFN11 mutants, we measured the levels of chromatin-bound SLFN11 (Figure 2B). As reported, both wild-type and E669Q SLFN11 were recruited to chromatin following CPT treatment. Likewise, the S753P SLFN11 mutant exhibited chromatin recruitment following CPT treatment. In contrast, the remaining SLFN11 mutants (K652E, K652D, S753D, and K652E/S753P) were not further recruited to chromatin following CPT treatment. The increased background levels of these mutants could be attributed to their higher expression levels relative to S753P SLFN11 (Figure 1C). To verify these findings, we performed an immunofluorescence analysis with pre-extraction, which enabled us to observe chromatin-bound proteins exclusively (Figures 2C and S4). As reported previously, wild-type SLFN11 was observed at both the nuclear periphery and inner nucleus, with the colocalization of RPA2, upon CPT treatment. The S753P SLFN11 mutant also exhibited patterns similar to those of wild-type SLFN11, but the other SLFN11 mutants (K652E, K652D, S753D, and K652E/S753P) did not. Collectively, these results indicated that the ssDNA binding site (K652) and the dephosphorylation of S753 within SLFN11 are critical for its functions related to chromatin binding, replication blockage, and drug sensitivity.

### Forced nuclear expression of Schlafen 13 cannot mimic the phenotypes of Schlafen 11

**Figure 3:**
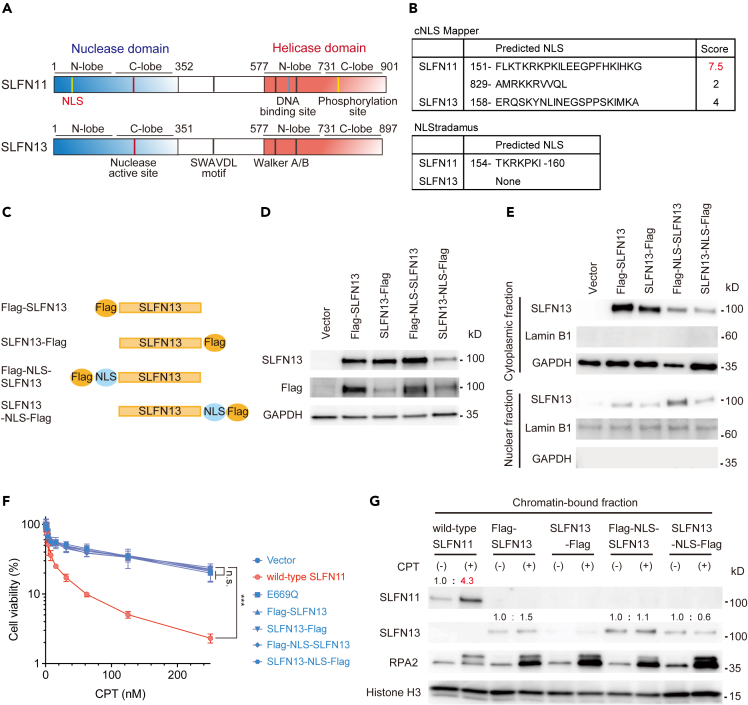
Forced expression of SLFN13 in the nucleus is not sufficient to confer SLFN11 functions on SLFN13 (A) Schematic diagram of the SLFN proteins. (B) Prediction of NLS signals in SLFN11 and SLFN13 with two algorithms (cNLS Mapper and NLStradamus). Scores of 7–8 indicate partial nuclear localization, 3–5 indicate localization in both the nucleus and cytoplasm, and 1–2 indicate cytoplasmic localization. (C) Schematics of the SLFN13 mutants generated in this study. (D and E) Blots of whole cell lysates (D) or cytoplasmic and nuclear fractions (E) from K562 cells expressing various constructs. The indicated antibodies were used. (F) Viability curves of the K562 cell lines. Experiments were performed and data were plotted as in Figure 1D. Error bars represent means ± standard deviations (n = 3). ∗∗∗p < 0.0001 (Dunnett test); n.s., not significant. (G) Blots of the chromatin-bound fraction of K562 cells expressing various constructs. Experiments were performed and data were plotted as in Figure 2B. See also Figure S5.

Having identified the crucial sites for SLFN11’s functions, we next explored the possibility of converting SLFN13 into SLFN11 by genetic modification. SLFN11 is dominantly expressed in the nucleus, as verified in various cells and tissues,^31^ while SLFN13 is localized in the cytoplasm.^28^ Through computational analyses using cNLS Mapper^32^ and NLStradamus,^33^ we identified a robust NLS at the N-terminus of SLFN11, in addition to the previously reported NLS at the C-terminus^22^ (Figures 3A, 3B, and S5A). Conversely, only a minimal NLS was detected in SLFN13 (Figures 3A, 3B, and S5A).

Because SLFN11 exerts its functions on chromatin (Figures 1 and 2), we attempted to overexpress SLFN13 in the nucleus. Accordingly, we constructed SLFN13-expression plasmids with a FLAG tag at the N-terminus (Flag-SLFN13) or the C-terminus (SLFN13-Flag) or with a FLAG tag and NLS at the N-terminus (NLS-SLFN13) or the C-terminus (SLFN13-NLS) (Figure 3C). We generated stable K562 cells, which normally have undetectable levels of endogenous SLFN13, expressing these SLFN13 constructs (Figures 3D and S5B). A fractionation analysis demonstrated that NLS-SLFN13 exhibited the highest expression in the nucleus (Figure 3E). Cellular viability assays showed that wild-type SLFN11, but none of the SLFN13-carrying cells, conferred sensitivity to CPT, even though the expression level of NLS-SLFN13 in the nuclear fraction exceeded that of wild-type SLFN11 (Figures 3F and S5C). Moreover, upon CPT treatment, SLFN11, but none of the modified SLFN13s, was recruited to chromatin (Figure 3G). These results indicated that nuclear-localized SLFN13 per se cannot mimic the drug sensitivity and chromatin binding capabilities of SLFN11.

### The single-stranded DNA binding site enables Schlafen 13 to recapitulate the functions of Schlafen 11

**Figure 4:**
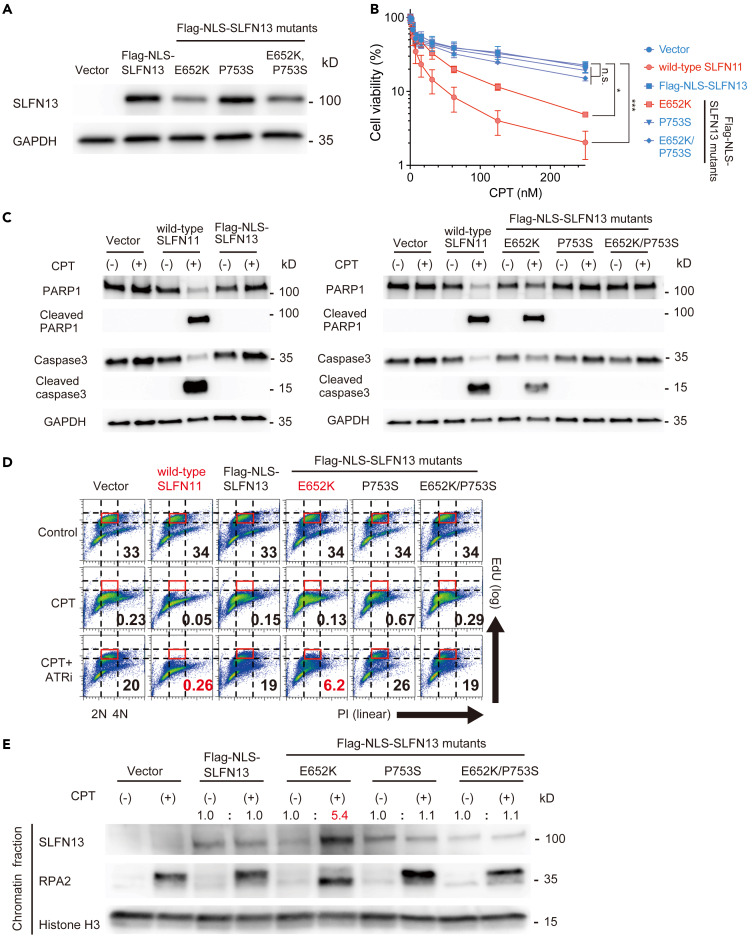
SLFN13 gains SLFN11-mimicking functions upon introducing the ssDNA binding site (E652K) but loses the functions if residue 753 is phosphorylatable (A) Blots of whole cell lysates from K562 cell lines expressing various constructs. The indicated antibodies were used. (B) Viability curves of K562 cell lines. Experiments were performed and data were plotted as in Figure 1D. Error bars represent means ± standard deviations (n = 3). ∗p < 0.05, ∗∗∗p < 0.0001 (Dunnett test); n.s., not significant. (C) Blots of whole cell lysates from K562 cell lines expressing various constructs after 250 nM CPT treatment for 24 h. The indicated antibodies were used. (D) Flow cytometry cell cycle data. Data are representative of two independent experiments. Experiments were performed and data were plotted as in Figure 2A. (E) Blots of the chromatin-bound fraction from K562 cells expressing various constructs. Experiments were performed and data were plotted as in Figure 2B. See also Figure S6.

Having found that K652E SLFN11 lost its chromatin-binding ability (Figures 1 and 2), we then introduced the E652K mutation into NLS-SLFN13. We also introduced the P753S mutation, mimicking the phosphorylation site of SLFN11 (S753), into NLS-SLFN13 and E652K NLS-SLFN13. We generated stable K562 cells expressing wild-type or mutant NLS-SLFN13 (Figures 4A and S6A). Viability assays revealed that the E652K NLS-SLFN13 had mild sensitivity to CPT, although the effect was not as strong as that of wild-type SLFN11 (Figure 4B). As expected, P753S NLS-SLFN13 did not show drug sensitivity (Figure 4B). Interestingly, E652K/P753S NLS-SLFN13 lost the acquired drug sensitivity of E652K NLS-SLFN13 (Figure 4B). The differences in cell death were morphologically validated by microscopy (Figure S6B) and immunoblotting against cleaved PARP1 and cleaved caspase 3 (Figure 4C). Cell cycle analysis revealed that E652K NLS-SLFN13 acquired the replication-block function, whereas E652K/P753S NLS-SLFN13 lost this capability (Figure 4D). Furthermore, E652K NLS-SLFN13 was recruited to chromatin following CPT treatment, whereas E652K/P753S NLS-SLFN13 was not (Figure 4E). These results indicated that NLS-SLFN13 can mimic the functions of SLFN11 when it acquires the ssDNA binding site (E652K). However, E652K NLS-SLFN13 loses these acquired functions if the amino acid residue at position 753 becomes phosphorylatable.

### Protein phosphatase 2A (PP2A) is a binding partner of Schlafen 11

**Figure 5:**
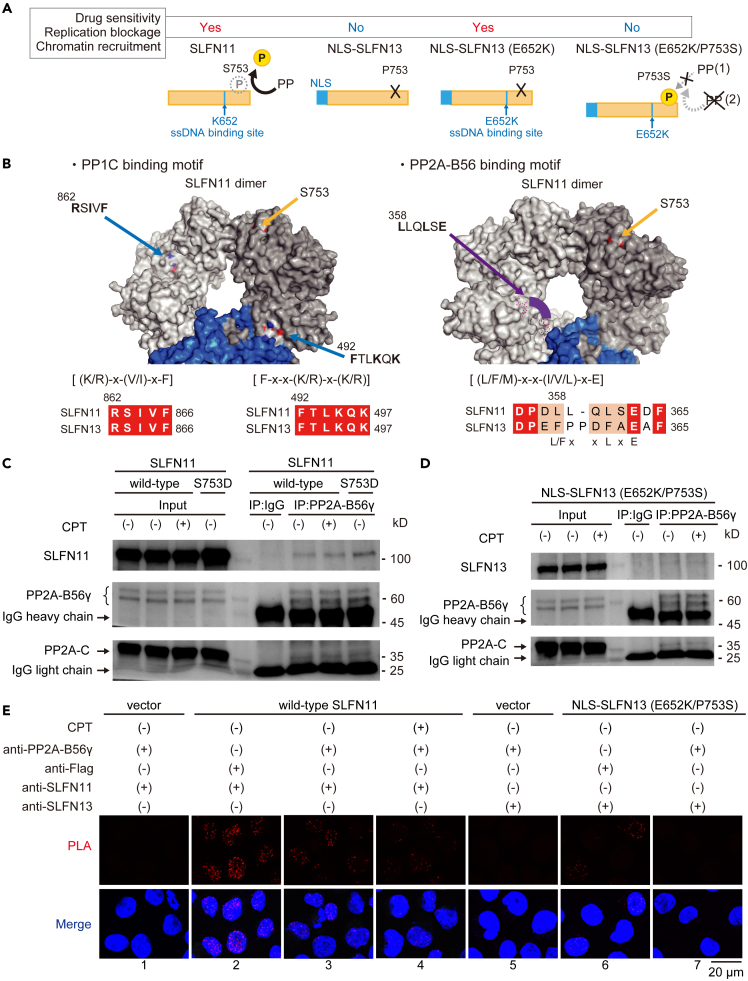
PP2A binds SLFN11 but probably not SLFN13 (A) The relationship between drug sensitivity enhancement and the presence of DNA binding or phosphorylation sites. Dephosphorylation of S753 in SLFN11 by a specific protein phosphatase (PP) is critical for enhanced drug sensitivity. This figure illustrates the hypotheses that NLS-SLFN13 (E652K/P753S) lacks a drug-sensitizing effect because either (1) the PPs for S753 in SLFN11 cannot access the protein as easily as they can in P753S SLFN13, or (2) SLFN11-selective PPs are present. (B) Three-dimensional surface structure of dimeric SLFN11 (PDB: 7ZEL), in which blue indicates the nuclease domain, and gray indicates the other domains. There are two short linear motifs (SLiMs) for PP1c and an SLiM for PP2A-B56 in the SLFN11 amino acid sequence. Yellow arrows indicate the location of S753, blue arrows indicate the SLiM locations for PP1c, and purple arrows indicate the SLiM location for PP2A-B56. The purple arches between the red dots in the figure on the right indicate disordered regions. Lower: Alignment of the sequences corresponding to the SLiMs in SLFN11 and SLFN13. (C) Co-immunoprecipitation (IP) of SLFN11 and endogenous PP2A-B56γ from wild-type and S753D SLFN11-expressing K562 cells by a PP2A-B56γ antibody (vs. mouse IgG control). Cells were treated with 100 nM CPT for 4 h. The PP2A-C subunit was used as a positive control for the interacting protein. IgG heavy and light chains were also detected at 50 and 25 kDa, respectively. (D) Co-IP of E652K/P753S SLFN13 mutant and endogenous PP2A-B56γ in E652K/P753S SLFN13-expressing K562 cells, detected by the same method as in panel C. (E) Representative images of PLA foci between PP2A-B56γ and SLFN11 or E652K/P753S SLFN13. Cells were treated with 100 nM CPT for 4 h. PLA in empty vector-expressing cells served as a negative control. PLA between Flag and SLFN11 or SLFN13 was used as a positive control. The scale bar represents 20 μm.

Since the P753S mutation nullified the chromatin-binding function in the E652K NLS-SLFN13 mutant, whereas SLFN11 harbors an intrinsic S753 site, we speculated that either (1) phosphatases for S753 in SLFN11 may be unable to access P753S in SLFN13 owing to differences in the local protein structure, or (2) SLFN11 may utilize specific phosphatases for S753 that are not functional with SLFN13 (Figure 5A).

To examine the former possibility, we generated two NLS-SLFN13 constructs with portions of the SLFN11 amino acid sequence, as illustrated in Figure S7A. The N-lobe chimera NLS-SLFN13 (E652K/P753S) contains the SLFN11 amino acid sequence from residues 577–731, while the C-lobe chimera NLS-SLFN13 (E652K/P753S) has the SLFN11 amino acid sequence from residues 731–901. Although we generated K562 cells stably overexpressing these plasmids, neither the N-lobe nor C-lobe mutant gained drug sensitivity (Figures S7B and S7C), indicating that the protein structure surrounding P753S in SLFN13 is not likely to cause the impaired dephosphorylation.

Then, to investigate the latter possibility, we performed a protein structure analysis focused on the short linear motif (SLiM) of protein phosphatases (PPs).^34^ As the PP1 catalytic subunit γ (PPP1CC) reportedly binds SLFN11 and dephosphorylates three phosphorylation sites (S219, T230, and S753), we found two SLiMs of the PP1 catalytic subunit (PP1C) [(K/R)-x-(V/I)-x-F] and [F-x-x-(K/R)-x-(K/R)] in both SLFN11 and SLFN13 (Figure 5B).^35^^,^^36^ The two motifs were localized on the same surface of the dimerized SLFN11 (S753), implying that PP1 can access SLFN11 (S753). Furthermore, we determined that SLFN11, but not SLFN13, contains a SLiM for the PP2A regulatory B56 subunit (PP2A-B56) [(L/F/M)-x-x-(I/V/L)-x-E] on this surface (Figure 5B).^37^ This region is annotated as a disordered region, suggesting the presence of a binding protein.

To evaluate the interaction between PP2A-B56 and SLFN11, we conducted a co-immunoprecipiation assay using the PP2A-B56γ antibody, a well-studied PP2A-B56 subtype.^38^ Wild-type SLFN11 and PP2A-C (another subunit of PP2A) both co-immunoprecipitated with PP2A-B56γ regardless of CPT treatment (Figure 5C). The interaction between SLFN11 and PP2A-B56γ was slightly enhanced in S753D SLFN11-overexpressing cells (Figure 5C). However, the interaction between PP2A-B56γ and SLFN13 was rather weak, while that between PP2A-B56γ and PP2A-C remained detectable (Figure 5D). We also evaluated these interactions by a proximity ligation assay (PLA). In SLFN11-negative cells (vector), no signals were detected with SLFN11 and PP2A-B56γ antibodies (Figure 5E1). As expected, SLFN11 and Flag antibodies produced signals in wild-type SLFN11- (Flag-tagged) expressing cells (Figure 5E2). The SLFN11 and PP2A-B56γ antibodies produced signals regardless of CPT treatment, consistent with the co-immunoprecipitation results in Figure 5C (Figures 5E3 and 5E4). In the SLFN13 negative cells (vector), no signals were detected with SLFN13 and PP2A-B56γ antibodies (Figure 5E5). However, antibodies for SLFN13 and Flag produced signals in the E652K/P753S SLFN13- (Flag-tagged) expressing cells (Figure 5E6), whereas SLFN13 and PP2A-B56γ antibodies did not produce signals in these cells. These results suggested that PP2A is a binding partner of SLFN11, but probably not of SLFN13.

### The protein phosphatase 2A binding motif in Schlafen 11 contributes to the Schlafen 11-dependent functions

**Figure 6:**
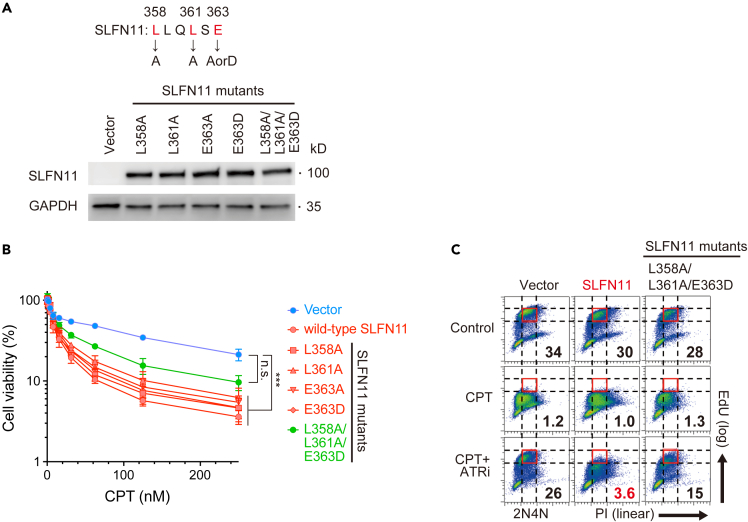
Putative PP2A binding motif contributes to the functions of SLFN11 (A) Upper: The SLFN11 mutation strategy. Lower: Western blots of the SLFN11 mutants. (B) Viability curves of various K562 cell lines. Experiments were performed and data were plotted as in Figure 1D. Error bars represent means ± standard deviations (n = 3). ∗∗∗p < 0.0001 (Dunnett test); n.s., not significant. (C) Cell cycle flow cytometry data. Data are representative of two independent experiments. Experiments were performed and data were plotted as in Figure 2A.

To examine the effect of the PPA2-B56 binding motif, we introduced single or multiple point mutations (L358A, L361A, E363A, E363D and L358A/L361A/E363D) within the motif and obtained cell lines overexpressing these SLFN11 mutants (Figure 6A). In cell viability assays, the triple L358A/L361A/E363D SLFN11 mutant attenuated drug sensitivity, compared to wild-type SLFN11 and the other single SLFN11 mutants (Figure 6B). Moreover, the triple L358A/L361A/E363D SLFN11 mutant showed reduced replication-blocking capability, in contrast to wild-type SLFN11 (Figure 6C). These results indicated that the PP2A-B56 binding motif is a regulatory site for the SLFN11-dependent drug sensitivity and replication blockage.

## Discussion

This is the first study to demonstrate that the ssDNA binding site (K652) and dephosphorylation at S753 in SLFN11 are critical for chromatin binding, replication blockage, and drug sensitivity in human cells. We also revealed that nuclear SLFN13 can mimic the functions of SLFN11, as long as SLFN13 possesses the ssDNA binding site (E652K). Moreover, we clarified the interaction between PP2A and SLFN11, and identified the putative PP2A binding motif as a regulatory domain of SLFN11. Our study provides important insights into the unique functions and characteristics of SLFN11.

### Do mice possess “*Slfn11*”?

The establishment of a mouse model for SLFN11 is an important stride toward the development of this target in clinical and basic sciences, particularly because SLFN11 has attracted attention as a candidate for overcoming drug resistance. Mice express several *Slfn* genes, including *Slfn1*, *-2*, *-3*, *-4*, *-5*, *-8*, *-9*, and *-10*, with only *Slfn5* being a shared gene between humans and mice.^39^ An ortholog of human SLFN11 has not been identified in the mouse genome. According to the phylogeny of *Slfn* genes based on a Bayesian analysis of amino acid sequences, mouse *Slfn8* and *Slfn9* are closer to each other and to human *SLFN13* than to human *SLFN11*.^20^ Hence, it has been assumed that there is no “*Slfn11*” in mice. However, we successfully converted SLFN13 into a protein resembling SLFN11 by introducing an NLS and an ssDNA binding site (K652) (Figure 3). Based on our findings and considering the additional functional sites and domains of SLFN11, we explored the possibility that mouse Slfn8/Slfn9 may exhibit SLFN11-like characteristics (Table S1). The mouse Slfn8/Slfn9 proteins are localized in the nucleus,^40^ and the phosphorylation site corresponding to SLFN11 (S753) is proline (P). Hence, mouse Slfn8/Slfn9 are likely to be constitutively active. The ssDNA binding site corresponding to SLFN11 (K652) is lysine (K) in mouse Slfn8/Slfn9, and thus mouse Slfn8/Slfn9 are likely to be capable of binding ssDNA. Just timely, during the review process, Alvi et al. reported that the mouse *Slfn8* and *Slfn9* genes share orthologous function with human *SLFN11*.^41^

### How does human Schlafen 11 exhibit its uniqueness?

Our study revealed that the ssDNA binding ability is required for the unique functions of SLFN11. Human SLFN13 and SLFN12 lack the SLFN11 (K652)-relevant ssDNA binding site,^42^^,^^43^ while SLFN5 harbors this site and the SLFN11 (S753)-relevant site is proline (P), and shows potent ssDNA and dsDNA binding ability (Table S1). However, the DNA-binding ability is related to the N-terminus, and the protein lacks dimerization sites (Table S1).^26^ Human SLFN14 shares 45% amino acid identity with SLFN11 (Table S2). Human SLFN14 harbors an SLFN11-relevant ssDNA binding site and the SLFN11 (S753)-relevant site is proline (P) (Table S1). Hence, SLFN14 could potently mimic SLFN11. However, SLFN14 expression is very low across ∼700 cell lines in the GDSC database (https://discover.nci.nih.gov/rsconnect/cellminercdb/), and thus its functions may have been overlooked.

We found that functionally active SLFN11 or SLFN13 showed lower levels of overexpression compared to their nonfunctional counterparts (Figures 1C and 4A). Nevertheless, cells overexpressing S753P SLFN11 remained viable without apparent phenotypic changes, suggesting that cells maintain the constitutively active SLFN11 below a certain level (Figure 1). Because S753D SLFN11 lost its SLFN11 function, dephosphorylation at S753 is apparently necessary to activate SLFN11 (Figure 1). Interestingly, this potential phosphorylation site is exclusive to primates (macaques, chimpanzees, and humans) (Table S1), implying that primates have acquired an SLFN11 ON/OFF switching system with the S753 site. We speculate that constitutively active SLFN11 might be harmful under specific conditions. For example, during B cell development SLFN11 expression is downregulated at the germinal center, where DNA damage and high proliferation occur.^44^ Additionally, highly proliferative embryonic stem cells turn off SLFN11 expression, whereas hematopoietic stem cells with lower proliferation rates express elevated levels of SLFN11.^45^ Under such highly proliferative conditions, constitutively active SLFN11 could be harmful. To control the switching system, SLFN11 must employ specific kinases and/or phosphatases, possibly including PP2A (Figure 5). Consistent with these findings, we demonstrated that the SLiM sequence of PP2A-B56 is conserved exclusively in primates (macaques, chimpanzees, and humans) and hedgehogs (Table S1). Interestingly, only human SLFN13, but no other species of SLFN13 (even in macaques or chimpanzees), lacks the DNA binding site (K652) (Table S1). If human SLFN13 had retained K652, it would likely function similarly to SLFN11. Strikingly, a most recent article revealed that *Caenorhabditis elegans* conserves SLFN-like domains.^46^ Accordingly, the evolution of the SLFN family may need to be revised based on functional sites and domains.

### Limitations of the study

In this study, we exclusively utilized a single cell line, K562. Other cell lines or cellular systems should also be assessed to confirm our findings.

## STAR★Methods

### Key resources table

**Table:** 

REAGENT or RESOURCE	SOURCE	IDENTIFIER
**Antibodies**		

Mouse monoclonal anti-SLFN11 (D-2) antibody	Santa Cruz Biotechnology	Cat#sc-515071
Human SLFN11 monoclonal antibody (5-14.12)	mAbProtein	Cat#R-S-001
Rabbit monoclonal anti-SLFN11 (D8W1B) antibody	Cell Signaling Technology	Cat#34858 RRID: AB_2799063
Mouse monoclonal anti-Glyceraldehyde-3-Phosphate Dehydrogenase (D4C6R) antibody	Cell Signaling Technology	Cat#97166S RRID: AB_2756824
Rabbit monoclonal anti-RPA32/RPA2 (E8X5P) XP antibody	Cell Signaling Technology	Cat#35869S RRID: AB_2799086
Rabbit polyclonal anti-SLFN13 antibody	Novus Biologicals	Cat#NBP1-93879 RRID: AB_11005330
Mouse monoclonal anti-FLAG M2 antibody	Sigma-Aldrich	Cat#F1804 RRID: AB_262044
Goat polyclonal anti-Lamin B1 (M-20) antibody	Santa Cruz Biotechnology	Cat#sc-6217 RRID: AB_648158
Rabbit monoclonal anti-PARP (46D11) antibody	Cell Signaling Technology	Cat#9532 RRID: AB_659884
Rabbit monoclonal anti-Cleaved PARP (Asp214) (D64E10) XP antibody	Cell Signaling Technology	Cat#5625 RRID: AB_10699459
Rabbit monoclonal anti-Caspase-3 (D3R6Y) antibody	Cell Signaling Technology	Cat#14220 RRID: AB_2798429
Rabbit monoclonal anti-Cleaved Caspase-3 (Asp175) (5A1E) antibody	Cell Signaling Technology	Cat#9664 RRID: AB_2070042
Rabbit polyclonal anti-Histone H3 antibody	Sigma-Aldrich	Cat#07-690 RRID: AB_417398
Mouse monoclonal anti-PP2A-B56γ (E-6) antibody	Santa Cruz Biotechnology	Cat#sc-374380 RRID: AB_10989746
Mouse monoclonal anti-PP2A-Cα/β (1D6) antibody	Santa Cruz Biotechnology	Cat#sc-80665 RRID: AB_1128770
Horse polyclonal anti-mouse IgG, horseradish peroxidase-linked antibody	Cell Signaling Technology	Cat#7076S RRID: AB_330924
Goat polyclonal anti-rabbit IgG, horseradish peroxidase-linked antibody	Cell Signaling Technology	Cat#7074P2 RRID: AB_2099233
Mouse monoclonal anti-goat IgG horseradish peroxidase-linked antibody	Santa Cruz Technology	Cat#sc-2354 RRID: AB_628490
Goat anti-Mouse IgG (H+L) Cross-Adsorbed Secondary Antibody, Alexa Fluor™ 488	Invitrogen	Cat#A-11001 RRID: AB_2534069
Goat anti-Mouse IgG (H+L) Cross-Adsorbed Secondary Antibody, Alexa Fluor™ 568	Invitrogen	Cat#A-11011 RRID: AB_143157

**Chemicals, peptides, and recombinant proteins**		

Camptothecin (CPT)	Cayman Chemical	Cat#11694
Ceralasertib (AZD6738, ATR inhibitor)	MedChemExpress	Cat#HY-19323
Puromycin	InvivoGen	Cat#ant-pr-1
RPMI 1640 medium with L-Gln, liquid	Nacalai Tesque	Cat#30264-85
Fetal bovine serum	Biowest	Cat#S1810-500
Antibiotic-Antimycotic Mixed Stock Solution (100x)	Nacalai Tesque	Cat#09366-44
RIPA buffer	Nacalai Tesque	Cat#16488-34
Protease inhibitor cocktail for Use with Mammalian Cell and Tissue Extracts	Nacalai Tesque	Cat#25955-11
Benzonase nuclease	Miilipore	Cat#E1014-25KU
Sample buffer solution with 2-ME (2x) for SDS-PAGE	Nacalai Tesque	Cat#30566-22
Bovine serum albumin (BSA)	Wako	Cat#015-21274
Clarify Max Western ECL Substrate	Bio-Rad Laboratories	Cat#1705062
4% paraformaldehyde	Nacalai Tesque	Cat#09154-56
Mounting Medium with DAPI	Abcam	Cat#ab104139

**Critical commercial assays**		

In-Fusion Snap Assembly Master Kit	Clontech	Cat#638947
KOD plus Mutagenesis Kit	TOYOBO	Cat#SMK-101
Subcellular Protein Fractionation Kit	Thermo Scientific	Cat#78840
Trans-Blot Turbo RTA Transfer Kit	Bio-Rad Laboratories	Cat#1704272
Pierce Protein A/G Magnetic Beads	Thermo Scientific	Cat#88802
ATPlite 1-step kit	PerkinElmer	Cat#6016731
Click-iT™ Plus EdU Alexa Fluor™ 488 Flow Cytometry Assay Kit	Invitrogen	Cat#C10632
Duolink *In Situ* PLA probe anti-Mouse PLUS	Sigma-Aldrich	Cat#DUO92001-30RXN
Duolink *In Situ* PLA probe anti-Rabbit MINUS	Sigma-Aldrich	Cat#DUO92004-30RXN
Duolink *In Situ* Detection Reagents Red	Sigma-Aldrich	Cat#DUO92008-30RXN
Duolink *In Situ* Wash buffer, Fluorescence	Sigma-Aldrich	Cat#DUO82049-4L

**Deposited data**		

Experimental Data	This paper	Mendeley Data https://doi.org/10.17632/tcsj29mr2h.1

**Experimental models: Cell lines**		

Human: leukemia K-562 cells	Developmental Therapeutics Program (NCI/NIH)	N/A

**Oligonucleotides**		

Forward primer to amplify repeat cassette sequences of the piggyBac system CCGCTC GAGTTAACCCTAGAAAGATAATCATATTGT GACGTACGTTAAAGATAATCATGCGTAAA ATTGACGCATGTTCGAAATGCATGG	Eurofins	N/A
Reverse primer to amplify repeat cassette sequences of the piggyBac system CCGCT CGAGTTAACCCTAGAAAGATAGTCTGCG TAAAATTGACGCATGCGAATTCGGTACC ATGCATTTCGAACATGCG	Eurofins	N/A
Forward primer to amplify 3xFL-IRES-PuroR-HSV TK poly(A) signal fragment CGGAATTC ATGGGCGTTGCCATGCCAGGTGCCGAAG ATGATGTGGTGTAACAATTCATGGACTAC AAAGACCATGACGG	Eurofins	N/A
Reverse primer to amplify 3xFL-IRES-PuroR-HSV TK poly(A) signal fragment ACATGCA TGCGAACAAACGACCCAACACCGTGCG TTTTATTCTGTCTTTTTATTGCCGGTCGAC TCAGGCACCGGGCTTGCGGG	Eurofins	N/A
Primers to amplify SLFN mutants, see Table S3	Eurofins	N/A

**Recombinant DNA**		

pCAG2-hyPB	Moribe et al.^44^	N/A
pPCIP vector	Moribe et al.^44^	N/A

**Software and algorithms**		

Image J ver.1.54f	NIH	https://imagej.net/
GraphPad Prism 7	GraphPad	https://www.graphpad.com/
FACSDiva	BD Biosciences	https://www.bdbiosciences.com/ja-jp/products/software/instrument-software/bd-facsdiva-software
CellMiner CDB (web application for analysis of NCI-60 database)	Luna et al.^47^	https://discover.nci.nih.gov/rsconnect/cellminercdb/
Clustal Omega by European Bioinformatics Institute (EMBL-EBI) search	Madeira et al.^48^ Sievers et al.^49^	https://www.ebi.ac.uk/Tools/msa/clustalo/
cNLS Mapper	Kosugi S. et al.^38^	http://nls-mapper.iab.keio.ac.jp/cgi-bin/NLS_Mapper_form.cgi
NLStradamus	Nguyen Ba A.N. et al.^39^	http://www.moseslab.csb.utoronto.ca/NLStradamus/

### Resource availability

#### Lead contact

Requests for further information and for resources or reagents should be directed to and will be filled by Junko Murai (muraij@ttck.keio.ac.jp).

#### Materials availability

Plasmids and cell lines generated in this study are available upon request.

#### Data and code availability

• All datasets generated in this study have been deposited at Mendeley Dataand are publicly available as of the date of publication. The DOI is listed in the key resources table.
• This paper does not report original code.
• Any additional information required to reanalyze the data reported in this paper is available from the lead contact upon request.

### Experimental model and study participant details

#### Cell lines

K562 (human) cells were obtained from the Developmental Therapeutics Program (NCI/NIH) and grown in RPMI 1640 (Nacalai Tesque) medium supplemented with 10% fetal bovine serum and 1% penicillin-streptomycin. Cells were cultured at 37°C in a 5% CO_2_ incubator. The PiggyBac system was used for stable gene expression in all cell lines.

### Method details

#### Generation of overexpressing cells

The expression vector containing the hyperactive PB transposase cDNA under the CAG promoter (pCAG2-hyPB) and the pPCIP vector were described previously.^44^ SLFN11 cDNA with a Flag tag (5′-GACTACAAGGACGACGATGACAAG-3′) added to the N-terminus and SLFN13 cDNA with a Flag tag and nuclear localization signal (NLS) sequence derived from SV40 (5′-CCAAAGAAGAAGCGGAAGGTC-3′) were amplified and integrated into the NotI and MluI sites of the pPCIP vector, using an In-Fusion Snap Assembly Master kit (Clontech). Point mutations in SLFN11 and SLFN13 expression vectors were generated using a KOD plus Mutagenesis Kit (TOYOBO), and validated by sequence analysis. The primers used for mutations are listed in the SI Appendix, Table S3. The PiggyBac system was used for stable gene expression in all cell lines. The pPCIP and pCAG2-hyPB vectors were cotransfected into K562 cells by electroporation. The cells were exposed to 2 μg/mL puromycin (InvivoGen) for 2 weeks, and surviving cells were used for assays.

#### Immunoblotting and quantification

To prepare whole cell lysates, cells were lysed with RIPA buffer (Nacalai Tesque) containing a protease inhibitor cocktail (Nacalai Tesque). After thorough mixing and incubation at 4°C for 30 min, lysates were centrifuged at 15,000 × g at 4°C for 10 min, and supernatants were collected. To prepare cytoplasmic, nuclear, and chromatin-bound fractions, we used a Subcellular Protein Fractionation Kit (Thermo Scientific) according to the manufacturer’s instructions. Samples were mixed with Sample Buffer Solution with 2-ME (2×) for SDS-PAGE (Nacalai Tesque), heated at 95°C for 10 min, and loaded onto Mini-PROTEAN TGX Gels (Bio-Rad Laboratories). The proteins on the gel were transferred to polyvinylidene difluoride membranes using a Trans-Blot Turbo RTA Transfer Kit (Bio-Rad Laboratories). Membranes were blocked with 4% bovine serum albumin (BSA; Wako) in phosphate-buffered saline (PBS) with 0.1% Tween-20 (PBS-T) for 1 h at room temperature, and immunoblotted at 4°C overnight with primary antibodies. The primary antibodies were diluted in 5% BSA/PBS-T at 1:2,500 for anti-SLFN11 (D-2; Santa Cruz Biotechnology), anti-SLFN11 (5-14.12; mAbProtein), anti-glyceraldehyde 3-phosphate dehydrogenase (Cell Signaling Technology), anti-RPA2 (Cell Signaling Technology), anti-SLFN13 (Novus Biologicals), anti-Flag (Sigma-Aldrich), anti-Lamin B1 (Santa Cruz Biotechnology), anti-PARP1 (Cell Signaling Technology), anti-cleaved PARP1 (Cell Signaling Technology), anti-caspase-3 (Cell Signaling Technology), and anti-cleaved caspase-3 (Cell Signaling Technology), and at 1:10,000 for anti-histone H3 (Sigma-Aldrich). After overnight incubation, membranes were incubated with horseradish peroxidase-conjugated secondary antibodies (Cell Signaling Technology, Santa Cruz Biotechnology). The secondary antibodies were diluted at 1:5,000 in 4% BSA PBS-T. Protein signals were visualized with Clarity Max Western ECL Substrate (Bio-Rad Laboratories) and an ImageQuant LAS 4000 mini (Cytiva) imager. Band intensity quantification was performed with the ImageJ software. The intensity of the background was subtracted, and the intensity of each control sample was set as 1. Signal intensity measurements were adjusted according to the signal intensity of histone H3, and the signal ratio was calculated.

#### Immunoprecipitation (IP)

Cells were untreated or treated with 100 nM CPT for 4 h. Paraformaldehyde (Nacalai Tesque) was added directly to the medium to 0.04% final concentration for cross-linking, and the cells were incubated at room temperature for 10 min. Glycine-NaOH (pH 7.5) was then added to 100 mM final concentration, and the cells were incubated at room temperature for 4 min. Cells were washed with PBS and lysed with 500 μL of NETN 50 buffer (50 mM NaCl, 50 mM Tris, pH 7.5, 0.1 mM EDTA, 1% NP40, supplemented with 1×protease inhibitor cocktail and 2 μL benzonase). After thorough mixing and incubation on ice for 40 min, the lysates were centrifuged at 16,000 ×g at 4°C for 10 min, and supernatants were collected. After the supernatants were transferred to a new tube, 2 μg of anti-PP2A-B56γ (E-6; Santa Cruz Biotechnology) was added, and the solutions were incubated at 4°C overnight with agitation. Meanwhile, 25 μL of Pierce protein A/G magnetic beads (Thermo Scientific) were placed in another tube and washed twice with NETN 50 buffer. The beads in NETN 50 buffer were added to the supernatants, and incubated for 1 h at 4°C. The beads were washed 5 times with NETN 50 buffer, and the IP samples were collected in 50 μL of Sample Buffer Solution with 2-ME (1×) for SDS-PAGE (Nacalai Tesque). The IP and input samples were then heated at 95°C for 20 min to reverse the cross-linking and analyzed by immunoblotting.

#### Cellular viability assay

Cells were plated in 384-well white plates (Greiner) at 4,000 cells/well in 40 μL complete medium with various concentrations of camptothecin (CPT; Cayman Chemical). Viability was determined using ATPlite 1-step kits (PerkinElmer). After 72 h, ATPlite solution was added (20 μL/well), and luminescence was then measured with an Infinite M200 Microplate Reader (TECAN). The ATP level in untreated cells was defined as 100%. The viability (%) of treated cells was defined as ATP-treated cells/untreated cells × 100. The data were transferred to the GraphPad Prism 7 software, and graphs were created.

#### Cell cycle analysis

Cells were incubated with 100 nM CPT and/or 1 μM ATR inhibitor (AZD6738; MedChemExpress) for 4 h. After an incubation with 10 μM 5-ethynyl-2′-deoxyuridine (EdU) for 1 h, cells were fixed with 4% paraformaldehyde (Nacalai Tesque) in PBS for 15 min. To detect the EdU signal, we used Click-iT Plus EdU Alexa Fluor 488 Flow Cytometry Assay Kits (Invitrogen), according to the manufacturer’s instructions. Propidium iodide (PI) was used to measure DNA content. Data were collected with a MACS Quant Analyzer 10 (Miltenyi Biotec) and analyzed with the BD FACSDiva software (BD Biosciences).

#### Immunofluorescence microscopy

Cells were deposited onto slide glasses (Superfrost Plus Microscope Slides precleaned; Fisher Scientific) by cytospin. The deposited cells were fixed with 4% paraformaldehyde (Nacalai Tesque) in PBS for 10 min, followed by permeabilization with 0.5% Triton-X 100/PBS for 15 min. To detect the chromatin-bound proteins, the deposited cells were pretreated with cold 0.5% Triton-X 100/PBS for 30 s on ice before fixation. After washing with PBS-T, the cells were incubated with 4% BSA/PBS-T for 10 min. The cells were then incubated at 4°C overnight with primary antibodies diluted in 4% BSA/PBS-T, at 1:2,500 for anti-Flag (Sigma-Aldrich), and 1:300 for anti-RPA2 (Cell Signaling Technology) and anti-SLFN11 (D-2; Santa Cruz Biotechnology). The cells were with PBS-T and then incubated for 2–4 h with appropriate secondary antibodies diluted 1:1,000 in 4% BSA/PBS-T. After washing with PBS-T, cells were mounted with Mounting Medium with DAPI (Abcam). Images were captured with a Zeiss LSM 900 confocal microscope. Slides were protected from light throughout the process.

#### Proximity-ligation assay (PLA)

The proximity-ligation assay was performed by using the Duolink *in situ* red starter kit (Sigma-Aldrich), according to the manufacturer’s instructions. Cells were fixed with 4% paraformaldehyde in PBS (Nacalai Tesque) at room temperature for 10 min, washed with PBS twice, and then permeabilized with 0.5% Triton X-100/PBS at room temperature for 15 min. After washing with PBS twice, the cells were incubated with Duolink Blocking solution at 37°C for 1 h. The cells were then stained at 37°C for 1 h with primary antibodies diluted in Duolink Antibody Diluent, at 1:300 for anti-SLFN13 (Novus Biologicals), and at 1:1,000 for anti-PP2A-B56-γ (E-6; Santa Cruz Biotechnology), anti-SLFN11 (D8W1B; Cell Signaling Technology), and anti-Flag (Sigma-Aldrich). After washing with wash buffer A twice, cells were incubated with PLUS and MINUS PLA probes at 37°C for 1 h. The cells were washed with wash buffer A twice, and then incubated with the ligation reaction at 37°C for 30 min. After washing with wash buffer A twice, the amplification reaction was performed at 37°C for 100 min. After washing with wash buffer B twice and 1:100 wash buffer B, cells were mounted with Mounting Medium with DAPI (Abcam).

#### Acquisition of RNA-seq and drug sensitivity data from cell line databases

The correlation analysis was performed using CellMinerCDB, from the National Cancer Institute’s Center for Cancer Research (https://discover.nci.nih.gov/rsconnect/cellminercdb/#references).^47^ The drug activity data were obtained from NCI-60 and the Cancer Cell Line Encyclopedia (CCLE; https://portals.broadinstitute.org/ccle).

#### Alignment analysis

The amino acid alignment was performed using analytical tools from the European Bioinformatics Institute (EMBL-EBI; https://www.ebi.ac.uk/ebisearch) in 2022.^48^ The Clustal Omega program was used to generate the multiple sequence alignment.^49^

#### NLS analysis

The NLS sequence was predicted using the cNLS Mapper program^32^ (http://nls-mapper.iab.keio.ac.jp/cgi-bin/NLS_Mapper_form.cgi) and NLStradamus^33^ (http://www.moseslab.csb.utoronto.ca/NLStradamus/). For the cNLS Mapper analysis, the cutoff score was set to 2.0, and the protein region searched for bipartite NLSs with long linkers was within the terminal 60 amino acid region. For the NLStradamus analysis, the cutoff score was set to 0.4, and 4 state hidden Markov models (HMMs) were used to predict the NLS.

### Quantification and statistical analysis

Statistical analyses were performed with the GraphPad Prism 7 software. Test methods are described in each figure legend. Dunnett’s test was used to compare the differences among multiple groups. Statistical significance was set at ∗p < 0.05. Additional details can be found in the figure legends.

## Supplemental information

Document S1. Figures S1–S7 and Table S3

Table S1. Multiple amino acid alignment analysis between SLFNs of various species, related to Figure 1 and discussion

Table S2. Percentages of amino acid identity between SLFNs of various species, related to Figure 1 and discussion
