# Supplementary material for: The crucial role of single-stranded DNA binding in enhancing sensitivity to DNA-damaging agents for Schlafen 11 and Schlafen 13
Source: iScience. 2023 Nov 23;26(12):108529. doi: 10.1016/j.isci.2023.108529 (PMC10730379; doi:10.1016/j.isci.2023.108529)
Supplement: Document S1. Figures S1–S7 and Table S3 [file mmc1.pdf]

iScience, Volume *26*

Supplemental information

The crucial role of single-stranded DNA binding in enhancing sensitivity to DNA-damaging agents for Schlafen 11 and Schlafen 13

Kohei Fujiwara, Masashi Maekawa, Yuki Iimori, Akane Ogawa, Takeshi Urano, Nobuaki Kono, Hiroyuki Takeda, Shigeki Higashiyama, Makoto Arita, and Junko Murai

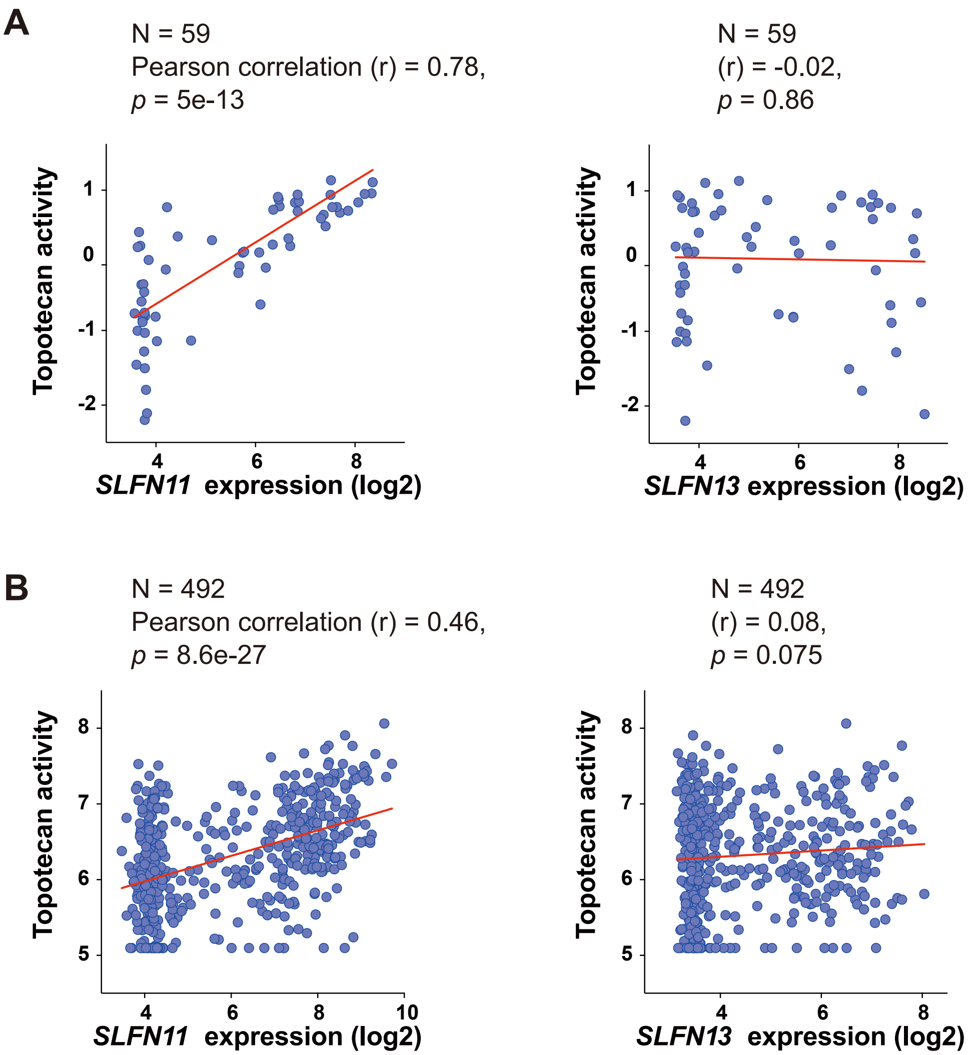

Figure S1. SLFN13 expression does not correlate with topotecan sensitivity, in contrast to SLFN11 expression, in independent databases, related to Figure 1.

(A)(B) Correlation analyses for topotecan activity and mRNA expression using cell line databases (A: NCI-60, B: CCLE-Broad-MIT). The number of samples (N), Pearson' s correlation coefficient (r), and p values are shown above each panel.

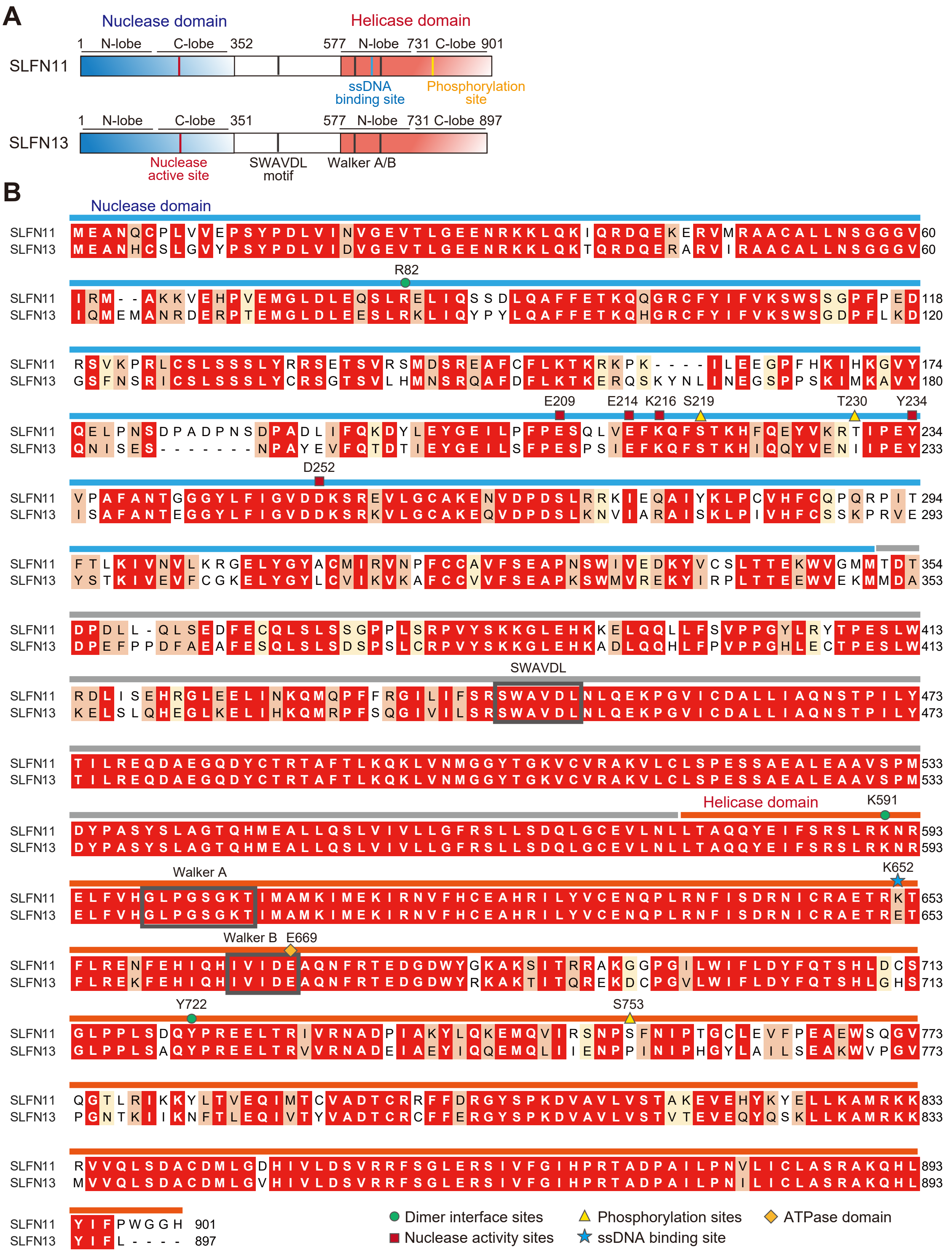

Figure S2. Differences in amino acid sequences between SLFN11 and SLFN13, related to Figure 1.

(A) Schematic diagrams of SLFN11 and SLFN13 proteins with the indicated key functional domains.

(B) Pairwise amino acid sequence alignment between SLFN11 and SLFN13, calculated using EMBL-EBI search and sequence analysis tools, including Clustal Omega. Residues are colored according to the percentage of identity (red = match, orange = more conserved, yellow = less conserved, white = mismatch). Nuclease domain, blue bar; linker domain, gray bar; and helicase domain, red bar.

Each functional site is highlighted in the corresponding color and shape.

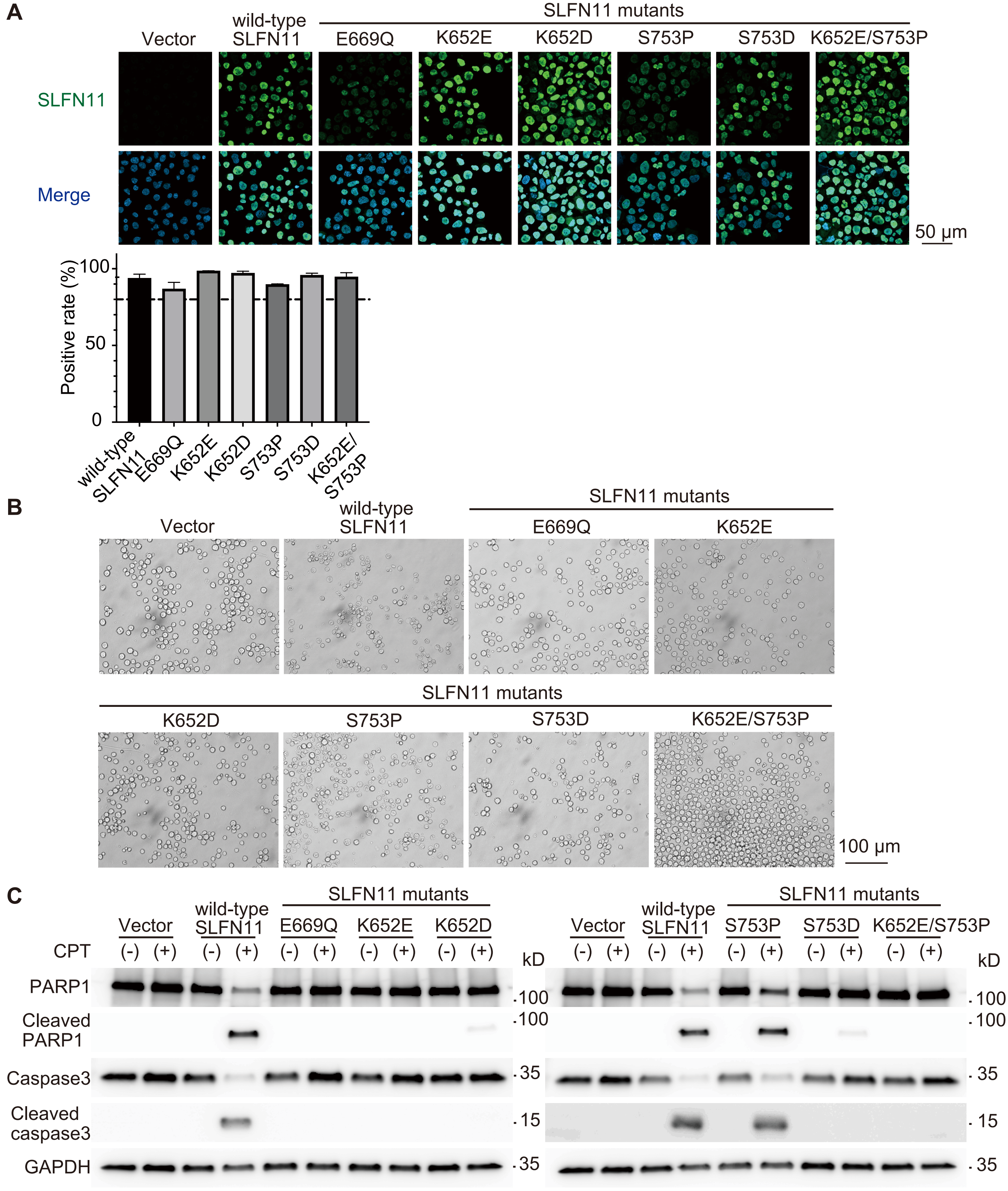

Figure S3. Apoptosis in responce to the DNA damage in SLFN11 mutants, related to Figure 1.

(A) Representative immunofluorescence images of cells showing positive expression of SLFN11 and SLFN11 mutants for the indicated K562 cell lines. Representative confocal microscopy images of SLFN11 (green) and DAPI (blue). The scale bar shows 50 μm. The right figure depicts the percentages of cells showing SLFN11 and SLFN11 mutant expression. The dotted line indicates 80%. Error bars represent means ± standard errors of the means (n = 3).

(B) Representative microscopy images for the indicated K562 cell lines after 24 h treatment with 250 nM camptothecin (CPT). The scale bar shows 100 μm.

(C) Protein expression levels of apoptosis markers after treatment with 250 nM CPT for 24 h.

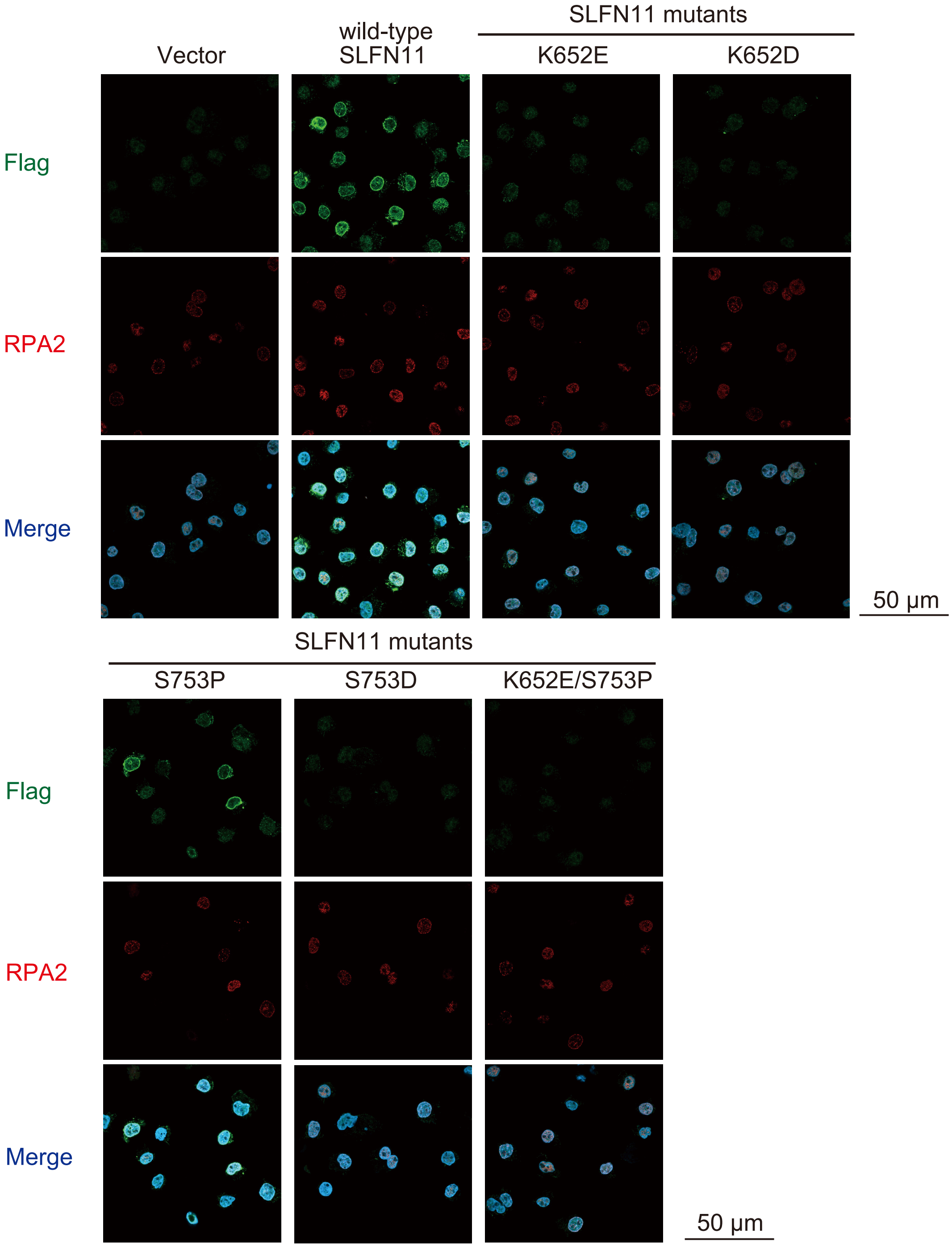

Figure S4. Chromatin binding in response to DNA damage in SLFN11 mutants, related to Figure 2. Immunofluorescence analyses of chromatin-bound proteins for the indicated K562 cell lines. Cells were treated with 100 nM CPT for 4 h. Representative confocal microscopy images for chromatin-bound Flag (green), RPA2 (red), and DAPI (blue). The scale bar shows 50 μm.

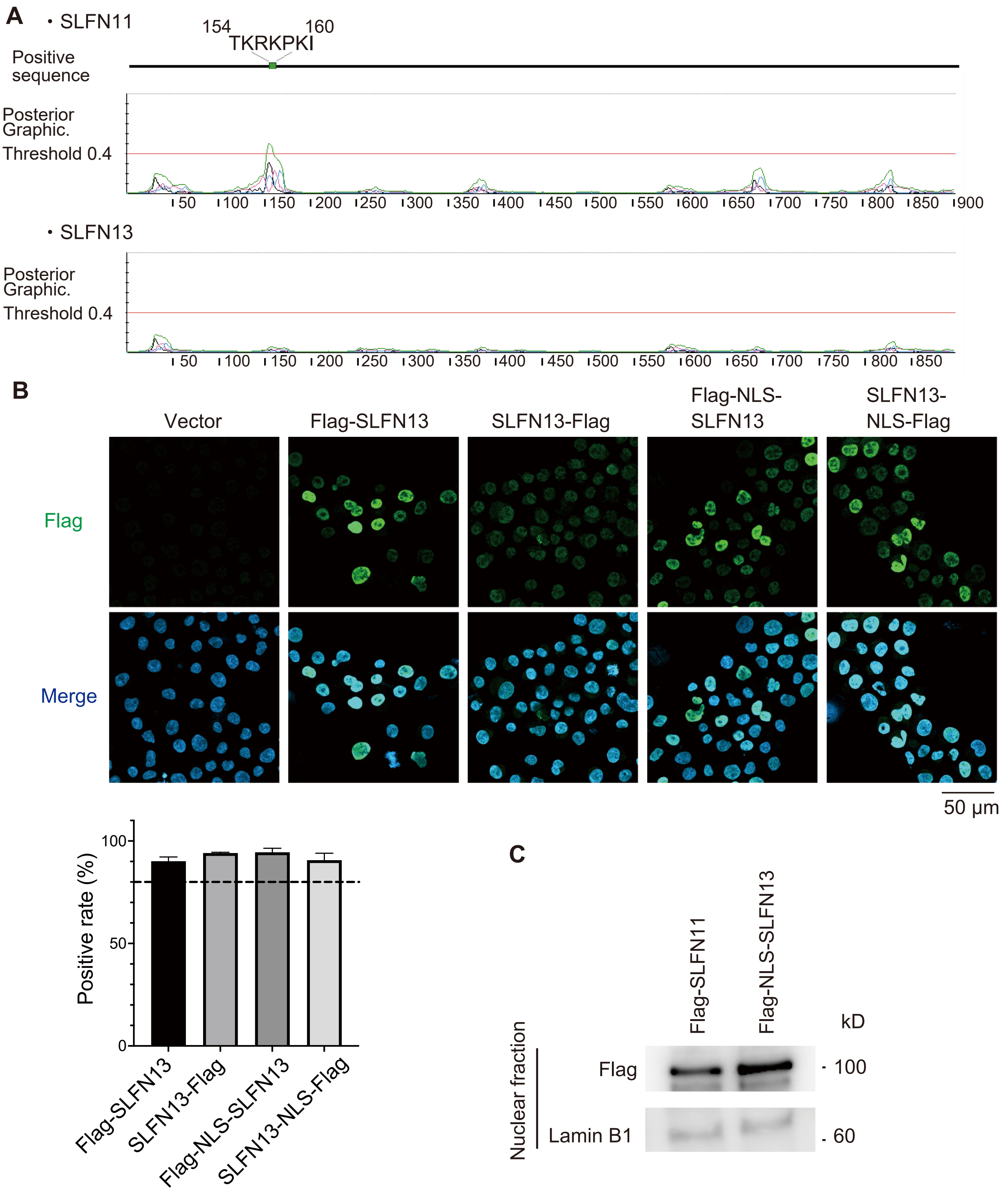

Figure S5. Expression of SLFN13 and SLFN13 mutants, related to Figure 3.

(A) Graphical representations of predicted NLS signals analyzed by NLStradamus. The graphs are based on static models of the four-state Hidden Markov Model. Red lines indicate the threshold value (0.4). The horizontal axis describes the amino acid sequence location, and the vertical axis describes the predicted NLS score. The upper positive sequence is an image with the NLS boxed, above a line representing the protein sequence.

(B) Representative immunofluorescence image of K562 cells expressing SLFN13. Representative confocal microscopy images for SLFN13 (green) and DAPI (blue). The scale bar shows 50 μm. The lower figure shows the percentages of cells positive for SLFN13 expression. The dotted line indicates 80%. Error bars represent means ± standard errors of the means (n = 3).

(C) Expression levels of Flag-tagged proteins in the nuclear fractions of SLFN11- and NLS-SLFN13-expressing K562 cell lines.

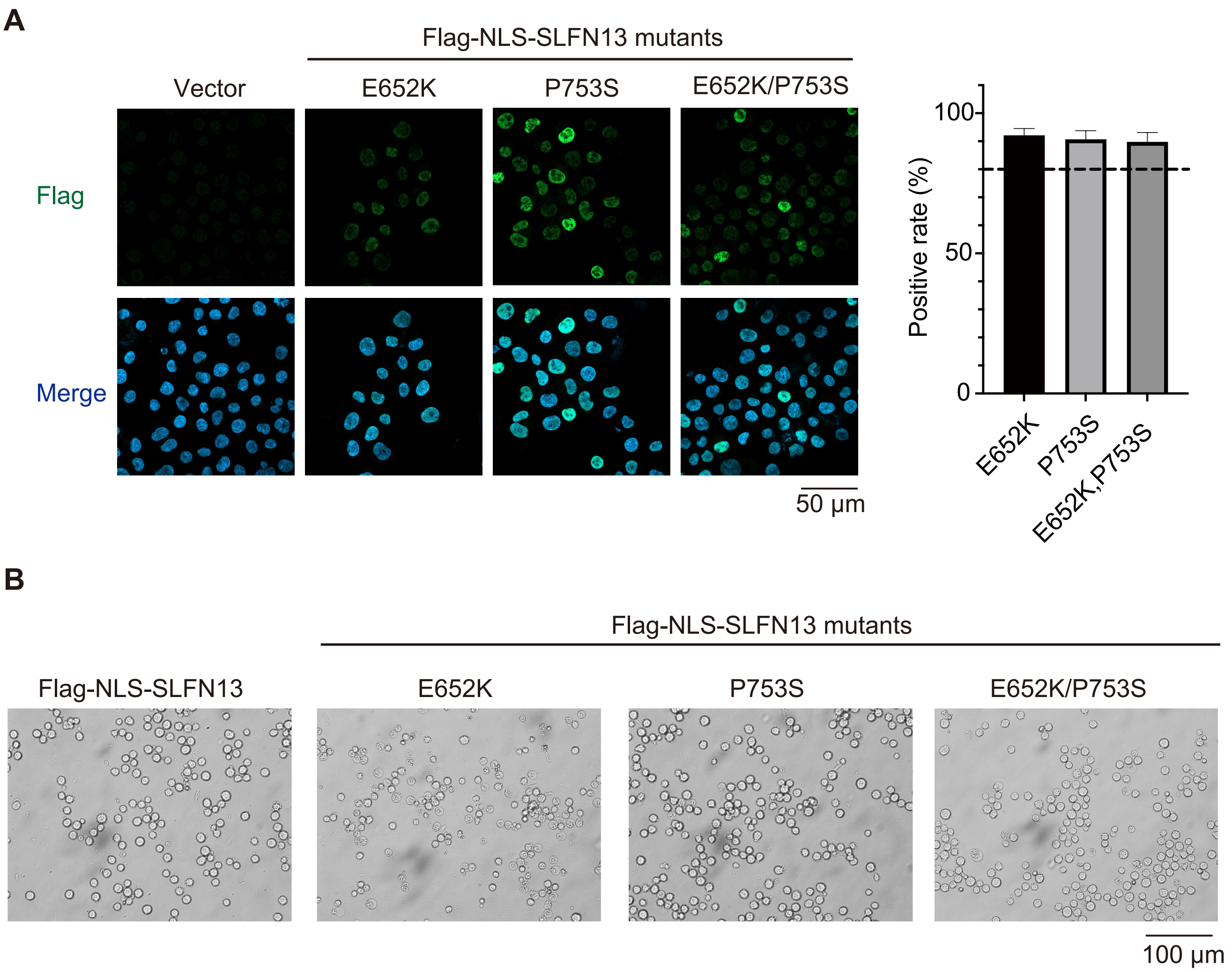

Figure S6. Expression and drug sensitivity in SLFN13 and SLFN13 mutants-expressing cell lines, related to Figure 4.

(A) Representative immunofluorescence image of K562 cells expressing SLFN13 mutants. Representative confocal microscopy images for SLFN13 (green) and DAPI (blue). The scale bar shows 50 μm. The lower figure shows the percentages of cells positive for SLFN13 mutant expression. The dotted line indicates 80%. Error bars represent means ± standard errors of the means (n = 3).

(B) Representative microscopy images of K562 cells after 24 h treatment with 250 nM camptothecin.

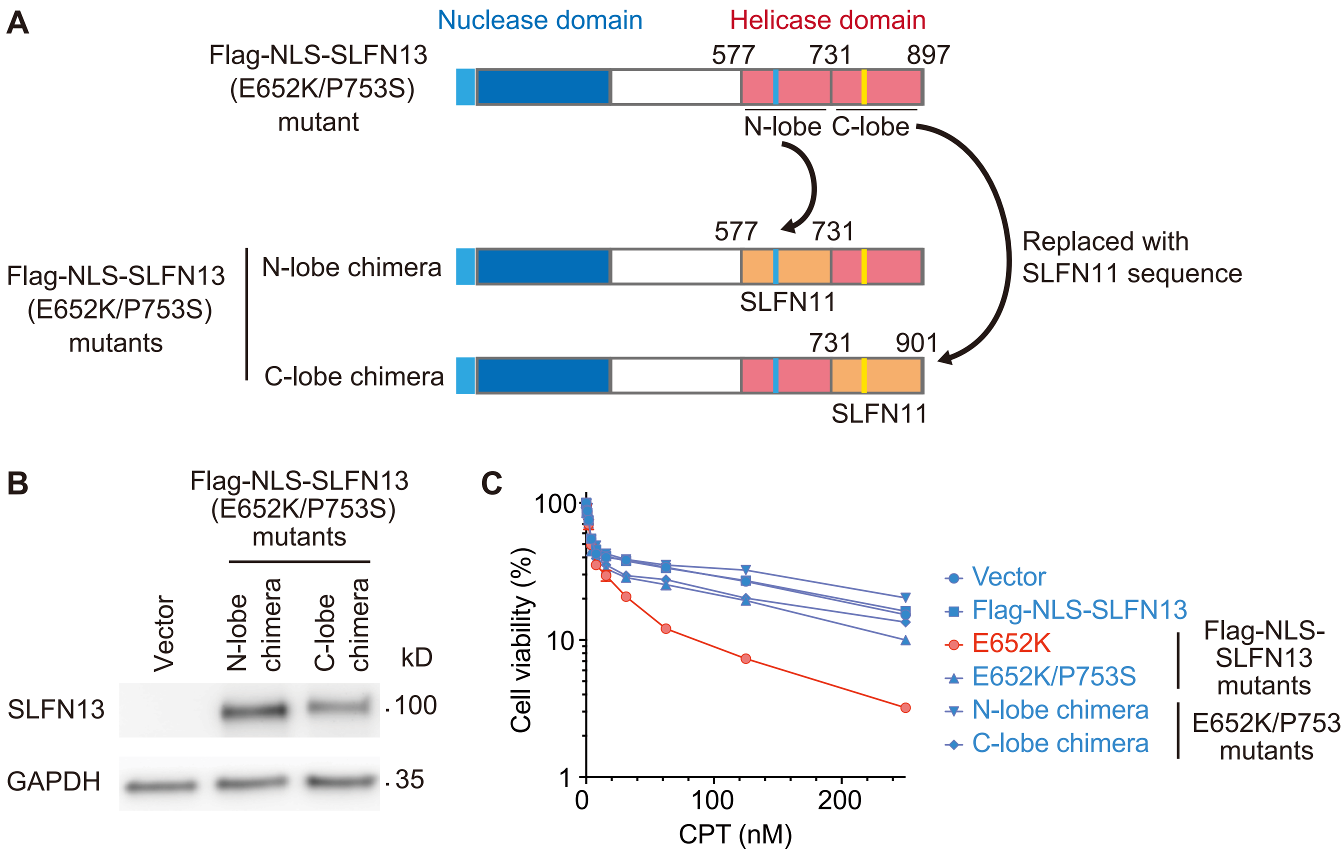

Figure S7. NLS-SLFN13 mutants (E652K/P753S) partially replaced with the SLFN11 amino acid sequence in the helicase domain do not gain drug sensitivity, related to Figure 5.

(A) Strategies for creating mutants of the helicase domain of the NLS-SLFN13 mutant (E652K/P753S). The N-lobe and C-lobe of the helicase domain of the NLS-SLFN13 mutant (E652K/P753S) were each replaced with the SLFN11 sequence. Blue box in the N-terminus, NLS; blue bar in the helicase domain, E652K site; and yellow bar in the helicase domain, P753S site.

(B) Protein expression levels of the indicated K562 cell lines, measured by western blotting.

(C) Drug resistance of the K562 cell lines. Cells were treated with various concentrations of camptothecin (CPT) for 72 h. Error bars represent means ± standard deviations (technical triplicates). Data are representative of two independent experiments.

Table S3. List of oligonucleotides (5' to 3'), related to STAR Methods Key resources table.

Primer design

Oligonucleotides		Sequence	Resource
FLAG-SLFN11	Fw	CTGGATCCAGCGGCCGCCACCATGGACTACAAGGACGACGATGACAAG	Eurofins Genomics
	Rv	CCATGAATTCACGCGCTAATGGCCACCCCACGG	
FLAG-SLFN13	Fw	CTGGATCCAGCGGCCGCCACCATGGACTACAAGGACGACGATGACAAGATGGAGGCAAATCACTGC	Eurofins Genomics
	Rv	CCATGAATTCACGCGTCACAGAAAAATATATAGGTGC	
SLFN13-FLAG	Fw	CTGGATCCAGCGGCCGCCACCATGGCAATGGAGGCAAATCACTGCT	Eurofins Genomics
	Rv	CCATGAATTCACGCGTCACTTGTCATCGTCGTCCTTGTAGTCCAGAAAAATATATAGGTGC	
FLAG-NLS-SLFN13	Fw	CTGGATCCAGCGGCCGCCACCATGGACTACAAGGACGACGATGACAAGCCAAAGAAGAAGCGGAAGGTC	Eurofins Genomics
	Rv	CCATGAATTCACGCGTCACAGAAAAATATATAGGTGC	
SLFN13-NLS-FLAG	Fw	CTGGATCCAGCGGCCGCCACCATGGCAATGGAGGCAAATCACTGCT	Eurofins Genomics
	Rv	CCATGAATTCACGCGTCACTTGTCATCGTCGTCCTTGTAGTCGACCTTCCGCTTCTTCTTTGG	
FLAG-SLFN11(E669Q)	Fw	GTCATTGACCAAGCTCAGAATTTCC	Eurofins Genomics
	Rv	GATGTGTTGAATGTGTTCAAAGTTTTCTC	
FLAG-SLFN11(K652E)	Fw	GAGACCCGGGAAACTTTCCTAAGAG	Eurofins Genomics
	Rv	TGCTCGGCAGATATTTCTATCACTG	
FLAG-SLFN11(K652D)	Fw	GAGACCCGGGACACTTTCCTAAGAG	Eurofins Genomics
	Rv	TGCTCGGCAGATATTTCTATCACTG	
FLAG-SLFN11(S753P)	Fw	ATCCTCCATTTAACATCCCCACTGG	Eurofins Genomics
	Rv	TACTTCTAATTACTTGCATTTCTTTTTG	
FLAG-SLFN11(S753D)	Fw	ATCCTGACTTTAACATCCCCACTGG	Eurofins Genomics
	Rv	TACTTCTAATTACTTGCATTTCTTTTTG	
FLAG-SLFN11(L358A)	Fw	GATCCAGATGCTCTACAGTTGTCTGAAGATTTTG	Eurofins Genomics
	Rv	TGTAGAGCATCTGGATCTGTGTCTGTCATCATGC	
FLAG-SLFN11(L361A)	Fw	CTACAGGCGTCTGAAGATTTTGAATGTCAGCTGAG	Eurofins Genomics
	Rv	TTCAGACGCCTGTAGAAGATCTGGATCTGTGTCTG	
FLAG-SLFN11(E363A)	Fw	CTTCTACAGTTGTCTGCAGATTTTGAATGTCAGC	Eurofins Genomics
	Rv	AGACAACTGTAGAAGATCTGGATCTGTGTCTGTC	
FLAG-SLFN11(E363D)	Fw	CTTCTACAGTTGTCTGATGATTTTGAATGTCAGC	Eurofins Genomics
	Rv	CAGACAACTGTAGAAGATCTGGATCTGTGTCTGTC	
FLAG-SLFN11	Fw	GCTCTACAGGCGTCTGATGATTTTGAATGTCAGC	Eurofins Genomics
(L358A/L361A/E363D)	Rv	CAGACGCCTGTAGAGCATCTGGATCTGTGTCTGTC	
FLAG-NLS-SLFN13 (E652K)	Fw	GAGACCCGGAAAACTTTCCTAAGAG	Eurofins Genomics
	Rv	TGCTCGGCAGATATTTCTATCACTG	
FLAG-NLS-SLFN13 (P753S)	Fw	ATCCTTCAATTAATATCCCCCATGG	Eurofins Genomics
	Rv	TTTCTATAATTAGTTGCATTTCTTGTTG	

To generate NLS-SLFN13 (E652K/P753S) N-lobe chimera

SLFN11(577-730)	Fw	AGGTTTTAAATCTGCTCACAGCCCAGCAGTATGAG	Eurofins Genomics
	Rv	ATCTGCATTGCGAACTATTCTGGTGAGCTCTTCTC	
pPCIP-FLAG-NLS-SLFN13	Fw	GTTCGCAATGCAGATGAAATAGCCGAGTAC	
(E652K/P753S, without 577-730)	Rv	CAGATTTAAAACCTCACAGCCGAGCTGGTCACTC	

To generate NLS-SLFN13 (E652K/P753S) C-lobe chimera

SLFN11(731-901)	Fw	CTCACCAGAGTAGTTCGCAATGCAGATCCAATAGC	Eurofins Genomics
	Rv	CCATGAATTCACGCGCTAATGGCCACCCCACGG	
pPCIP-FLAG-NLS-SLFN13	Fw	CGCGTGAATTCATGGACTACAAAGACCATGACGG	
(E652K/P753S, without 731-897)	Rv	TACTCTGGTGAGCTCTTCTCTTGGATACTGTGCTG	
